# Phylogenetic Analysis, Morphological Characteristics, and Cellular Tropism of Vaccine-Like Recombinant Strains of Lumpy Skin Disease Virus in China

**DOI:** 10.1155/tbed/2900359

**Published:** 2025-12-02

**Authors:** Zuxin Gong, Jinming Li, Yanli Zou, Jiaqi Dai, Shan Liu, Lin Li, Chunyan Feng, Fanqi Sun, Xin Li, Chenchen Liu, Zhiliang Wang, Gongguan Liu, Zhen Yang

**Affiliations:** ^1^Key Laboratory of Animal Diseases Diagnostic and Immunology, Ministry of Agriculture, MOE International Joint Collaborative Research Laboratory for Animal Health and Food Safety, The Belt and Road International Sci-Tech Innovation Institute of Transboundary Animal Disease Diagnosis and Immunization, College of Veterinary Medicine, Nanjing Agricultural University, Nanjing, China; ^2^China Animal Health and Epidemiology Center, Qingdao, China; ^3^Chinese Academy of Quality and Inspection and Testing, Beijing, China

**Keywords:** cellular tropism, heterologous vaccine, lumpy skin disease virus, morphogenesis, phylogeny analysis, vaccine-like recombinant strain

## Abstract

Lumpy skin disease virus (LSDV) causes lumpy skin disease (LSD), a highly contagious cattle disease that leads to substantial economic losses to the global cattle industry. Currently, it is imperative to further elucidate its biological characteristics and analyze the global epidemiological dynamics. In this study, two isolates of LSDV with genetic recombination were identified in Northern and Eastern China, which demonstrated broad host cell entry ability. Through electron microscopy, we further revealed its morphogenetic characteristics across its replication cycle for the first time. Viral particles sequentially formed crescent membranes, nucleoids, and lateral bodies, and ultimately developed into four types of mature virions: intracellular mature virions (IMVs), intracellular enveloped virions (IEVs), cell-associated enveloped virions (CEVs), and extracellular enveloped virions (EEVs). Whole-genome phylogenetic analysis revealed that both isolates belonged to Clade R4. Based on the global reference strains, our integrated analysis for temporal and geographical information revealed that LSDV has progressively expanded its endemic range, particularly in Asia, where recombinant Clade R4 strains have recently emerged as the predominant epidemic strains. Strikingly, recombination analysis detected a limited number of recombination signals between the LSDV isolates and goatpox virus (GTPV) or sheeppox virus (SPPV) strains, suggesting that the possibility of recombination between heterologous vaccines and LSDV cannot be fully excluded. These data may provide important information for prevention and control of LSD global outbreaks.

## 1. Introduction

Lumpy skin disease (LSD) is a subacute to acute infectious disease of cattle caused by LSD virus (LSDV). LSDV, together with goatpox virus (GTPV) and sheeppox virus (SPPV), belongs to the *Capripoxvirus* (CaPv) genus within the Poxviridae family, with a genome consisting of a large 151 kb double-stranded DNA [1]. Cross-border trade and arthropod vectors are the main pathways through which the disease is spread [2, 3]. Cattle are the primary susceptible hosts of LSDV, although certain wild animals may also contract the virus [4–6]. Clinical symptoms like fever, weight loss, decreased milk production, and reproductive dysfunction are frequently detected in affected cattle along with widespread skin nodules [7, 8]. Epidemiological data indicates an average morbidity rate of about 26% and a case fatality rate of about 7.5% [7]. Hence, LSD is an immediately notifiable animal disease, according to the World Organization for Animal Health (WOAH), because of its quick spread and detrimental effects on the cattle industry.

In 1929, LSD was first discovered in Zambia. As the epidemic persisted, the disease gradually spread from Africa to Europe and Asia [9]. However, the transmission of LSDV during this period did not attract widespread attention. It was not until 2017 that the first vaccine-recombinant strain was detected in Russia [10]. Subsequently, the outbreak rapidly expanded to Asian countries. In 2019, vaccine-like recombinant strain were identified in China and multiple countries across East and Southeast Asia [11–13]. Notably, the prevalent strains in South Asian nations such as India during the same period exhibited higher genomic homology with the KSGP-like strain from Kenya and LSDV field strains from Bangladesh [14], indicating potential multi-route transmission of LSD [13]. In China, LSD was first reported in Yili, Xinjiang, in 2019. Within 1 year, the disease rapidly expanded from the northwestern border regions to the southeastern provinces, including Anhui, Zhejiang, Guangdong, Fujian, and Jiangxi [15], and to northern provinces such as Inner Mongolia [16] and Heilongjiang [13]. Most of the outbreak strains in these regions have been identified as vaccine-like recombinant strains. However, the genomic information of the strain in Anhui Province remains undetermined. In addition, the sequence submitted from Inner Mongolia (GenBank: ON616408.1) is only a partial fragment, which limits comprehensive epidemiological investigations of LSD outbreaks in China.

Vaccination currently represents the most effective control and prevention measure for LSD. Based on their origins, existing LSD vaccines can be primarily categorized into homologous and heterologous vaccines. Studies have demonstrated that homologous live attenuated vaccines, represented by Neethling strain, can induce high levels of immune protection [17, 18]. However, these vaccines may cause adverse reactions such as local skin nodules, fever, and temporary reductions in milk production [19]. More critically, genetic recombination between homologous vaccine strains and field strains has led to the widespread emergence of vaccine-like recombinant strains in Asia, necessitating a reevaluation of the safety of homologous vaccines. GTPV and SPPV are the two other members of the CaPV genus, sharing high genomic homology with LSDV and exhibiting cross-protective immunity with LSDV. Leveraging this characteristic, heterologous vaccines derived from GTPV and SPPV have been employed for LSD prevention and control [20]. A recent study has linked these vaccine-like recombinant strains to mass vaccination campaigns in Kazakhstan using the Lumpivax vaccine. Although labeled as Neethling strain-based, subsequent genetic analyses revealed that Lumpivax appears to be a complex mixture containing multiple CaPVs (including GTPV) [21], raising concerns that heterologous vaccine strains may also contribute to recombination. Although heterologous vaccines are generally considered safer, there is currently a lack of analysis regarding potential recombination events between heterologous vaccine strains and LSDV strains.

Even though the continued spread of LSD, there are fewer LSDV whole-genome sequences available in public databases than the worldwide outbreak reports released by the WOAH. During the outbreak, the lack of genomic data may hinder the tracking of viral transmission and postpone the identification of novel variants. Furthermore, it is urgent to dissect the biological traits of LSDV, such as viral morphogenesis and cellular tropism. To address these gaps, this study updated genomic data for LSDV strains from outbreaks in Anhui and Inner Mongolia, further analyzed the cellular tropism of two isolates, provided the first complete description of the viral morphogenetic process, and evaluated the transmission dynamics and recombination patterns of global CaPV reference strains. These findings may provide new insights into the biological characteristics and epidemiology of LSDV.

## 2. Materials and Methods

### 2.1. Samples

A total of 10 and 12 skin nodule tissue samples, which tested positive for LSDV DNA by polymerase chain reaction (PCR) using primers (Primer F1/R1, Supporting Information 1: Table S3) targeting the LSDV DNA polymerase gene, were collected from Anhui Province in Eastern China and the Inner Mongolia Autonomous Region in Northern China in 2020, and preserved at the China Animal Health and Epidemiology Center.

### 2.2. Reagents

Dulbecco's modified Eagle's medium (DMEM) was purchased from Servicebio, and fetal bovine serum (FBS) from ExCell. Penicillin, streptomycin, bovine serum albumin (BSA), and a seamless cloning kit were purchased from Yeasen. DH5*α* competent cells were obtained from Tsingke, and an endotoxin-free plasmid extraction kit from Tiangen. TransIT-X2 transfection reagent was purchased from Mirus Bio (USA). Rabbit anti-LSDV polyclonal antibody was prepared in our laboratory, while Alexa Fluor 488-conjugated goat anti-rabbit IgG (H + L) was obtained from Thermo Fisher Scientific (USA), and 4′, 6-diamidino-2-phenylindole (DAPI) was purchased from Beyotime.

### 2.3. Cells

The Madin–Darby bovine kidney (MDBK) cells (ATCC CCL-22), MDCK (Canine kidney cells, ATCC CCL-34), CRFK (feline kidney cells, ATCC CCL-94), PK-15 (porcine kidney cells, ATCC CCL-33), 293T (human kidney cells, ATCC CRL-3216), A549 (human lung adenocarcinoma cells, ATCC CCL-185), HepG2 (human hepatocellular carcinoma cells, ATCC HB-8065), Vero (monkey kidney cells, ATCC CCL-81), and BHK-21 (hamster kidney cells, ATCC CCL-10) cell lines were kept in our laboratory. The LT (lamb testis cells) cell line was generously provided by Chunyan Feng (Chinese Academy of Quality and Inspection & Testing). The BTA-S2 (bovine dermal fibroblast cells) cell line was obtained from the National Infrastructure of Cell Line Resources. In accordance with the previously described strategy for virus isolation [13], MDBK cells were chosen for virus isolation. Nine different cell lines (MDCK, CRFK, PK-15, 293T, A549, HepG2, Vero, BHK-21, and BTA-S2) were used to evaluate the cellular tropism of LSDV. All cells were cultured in DMEM supplemented with 10% FBS for growth and maintained in DMEM with 3% FBS. Both the growth and maintenance media were supplemented with 100 U/mL penicillin and 100 µg/mL streptomycin. The cells were incubated at 37°C in a humidified atmosphere containing 5% CO_2_.

### 2.4. Virus Isolation

The skin nodule tissue samples were thoroughly ground after being mixed with DMEM. The supernatant was filtered through 0.45 μm filters and used for virus isolation after being centrifuged at 4000 rpm for 20 min at 4°C. In 24-well plates, MDBK cells were preseeded. The cell plates were shaken every 30 min, and the growth medium was swapped out for a mixture of 250 μL maintenance medium and 50 μL filtered supernatant for adsorption. After 2 h, the supernatant was replaced with 1 mL of maintenance medium. Cells were examined daily for cytopathic effect (CPE). The cell samples were frozen and then thawed three times on Day 5. MDBK cells were reinfected with the cell lysate and cultivated until CPE appeared.

### 2.5. Immunofluorescence Assay (IFA)

MDBK cells were seeded into 24-well plates and infected with either the isolates or phosphate-buffered saline (PBS) as a control. At 72 h post infection (hpi), the cells were fixed with 4% formaldehyde for 20 min, permeabilized with 0.5% Triton X-100 for 10 min, and blocked with 5% BSA for 2 h. Subsequently, the cells were incubated with rabbit anti-LSDV polyclonal antibody for 1 h, followed by incubation with Alexa Fluor 488-conjugated goat anti-rabbit IgG (H + L) for 1 h. Nuclei were counterstained with DAPI for 10 min. All steps were performed at room temperature, and the samples were washed three times with PBS after each step. Finally, the cells were examined using a fluorescence microscope.

### 2.6. Construction of the NMG01-mCherry Reporter Strain

The NMG01-mCherry reporter strain was constructed using a homologous recombination strategy [22]. In brief, the left homologous arms (Primer F2/R2, Supporting Information 1: Table S3) and right (Primer F3/R3, Supporting Information 1: Table S3) homologous arms were amplified by PCR from the regions flanking the *orf50* and *orf51* genes of NMG01 strain. The H5 promoter-mCherry expression cassette (Primer F4/R4, Supporting Information 1: Table S3) was synthesized by Tsingke Biotechnology (China). The backbone region of the pVAX1 plasmid (Thermo Fisher, USA) was amplified by PCR (Primer F5/R5, Supporting Information 1: Table S3). Finally, these four fragments were assembled using a seamless cloning kit to construct the donor plasmid. After sequence verification, the plasmid was transformed into DH5*α* competent cells and extracted using an endotoxin-free plasmid extraction kit. BHK-21 cells were transfected with the donor plasmid using TransIT-X2 transfection reagent, followed by infection with 1 MOI NMG01 strain after 24 h. At 96 hpi, the supernatant was replaced with sterile water (pH 7.4). After 10 min of incubation, the cells were vigorously vortexed for 2 min to induce osmotic lysis. The harvested viral suspension was then used to infect MDBK cells, followed by culturing in DMEM containing 1% methylcellulose for 96 h. NMG01-mCherry reporter strain was selected by screening for red fluorescent plaques under a fluorescence microscope. After three to five rounds of plaque purification, the NMG01-mCherry reporter strain was confirmed by PCR and Sanger sequencing.

### 2.7. Cell Tropism

Monolayers of MDCK, CRFK, PK-15, 293T, A549, HepG2, Vero, BHK-21, and BTA-S2 cells were infected with the NMG01-mCherry reporter strain at 0.1 multiplicity of infection (MOI) and incubated for 72 hpi, after which infected cells were harvested, subjected to three freeze–thaw cycles, followed by 0.45 μm filtration, and lysate from each cell line was used to inoculate fresh monolayers of the corresponding cell line for three subsequent passages (P1–P3). CPE on different cell lines was captured, and red fluorescence was observed by fluorescence microscopy at 72 hpi.

MDBK, MDCK, CRFK, PK-15, 293T, A549, HepG2, Vero, BHK-21, and BTA-S2 cell lines were infected with the NMG01-mCherry reporter strain at 0.01 multiplicity of infection (MOI) and adsorbed at 37°C for 2 h. Samples were collected at 0 hpi (immediately after adsorption), 24 hpi, 48 hpi, 72 hpi, and 96 hpi, followed by three freeze–thaw cycles. The lysates were centrifuged at 4000 × *g* for 10 min at 4°C, and the supernatants were harvested. Serially diluted viral supernatants were then inoculated onto MDBK cell monolayers in 48-well plates for 2 h. After adsorption, the inoculum was replaced with maintenance medium containing 1% methylcellulose. At 120 hpi, red fluorescent viral plaques were quantified by fluorescence microscopy.

### 2.8. Transmission Electron Microscopy (TEM)

MDBK cells were infected with the isolates at a MOI of 0.1. At 96 hpi, the cells were fixed with prechilled 2.5% glutaraldehyde solution and harvested using a cell scraper. Subsequently, the fixed samples were processed into ultrathin sections, and viral particles were visualized and imaged using HITACHI HT7800 transmission electron microscope.

### 2.9. Genome Sequencing

The DNA from the two isolates was extracted and subjected for next-generation sequencing (NGS) by the Beijing Genomics Institute (BGI). The sequencing data were assembled using the MEGAHIT v1.2.9 software. Gap regions in the assembled sequences were verified by PCR and Sanger sequencing. Finally, we obtained the complete genome sequences of strains NMG01 (GenBank ID: PQ682462.1) and AH01 (GenBank ID: PQ682461.1).

### 2.10. Phylogenetic Analysis

The sequences of two isolates and 190 reference sequences (Supporting Information 1: Table S1) obtained from the National Center for Biotechnology Information (NCBI) database were used in this study. In order to perform sequence alignment, MAFFT version 7.526 was used [23]. To infer the evolutionary history, the maximum likelihood (ML) method was employed. K3Pu + F + R3, the best-fit model, was chosen because it had the lowest Bayesian information criterion (BIC) score [24]. With IQ-TREE version 2.3.6, a phylogenetic tree was constructed. The bootstrap test with 1000 replicates is used to present the optimal tree [25]. An online platform for managing, displaying, annotating, and manipulating phylogenetic trees, Interactive Tree of Life (iTOL) v6 was used to visualize the phylogenetic tree [26].

### 2.11. Detection and Analysis of Recombination

The Recombination Detection Program (RDP, version 4.101) was employed to detect recombination events within the viral genome sequences analyzed in this study, which comprised 45 reference sequences and two sequences of isolates. The employed methodology involved aligning the sequences via MAFFT, followed by the application of four primary detection algorithms (RDP [27], GENECONV [28], MaxChi [29], and 3Seq [30]) to discern primary recombination signals. Subsequent to this, three supplementary algorithms (Chimaera [31], BootScan [32], and SiScan [33]) were integrated for the detection of secondary recombination signals. The *p*-value threshold was established at 0.01. Recombination signals that were identified by no fewer than three methods were considered potential recombination events following two rounds of detection, and a ML tree was constructed from the aligned sequences after excising the recombination regions. The SimPlot software version 3.5.1 was used to further characterize the potential recombinant events [34]. Based on the breakpoint positions of the 69 potential recombination events predicted by the RDP software, all recombination regions within each clade were considered, with overlapping regions counted only once, to calculate the proportion of the genome occupied by recombination regions in that clade. According to the clade annotations in the phylogenetic tree, the progeny strains identified in the 69 potential recombination events predicted by RDP were classified by clade. For each recombination event, strains belonging to the same clade were counted only once, thereby determining the number of recombination events contained within each clade.

## 3. Results

### 3.1. Cell Tropism of Emerging LSDV Isolates

**Figure 1:**
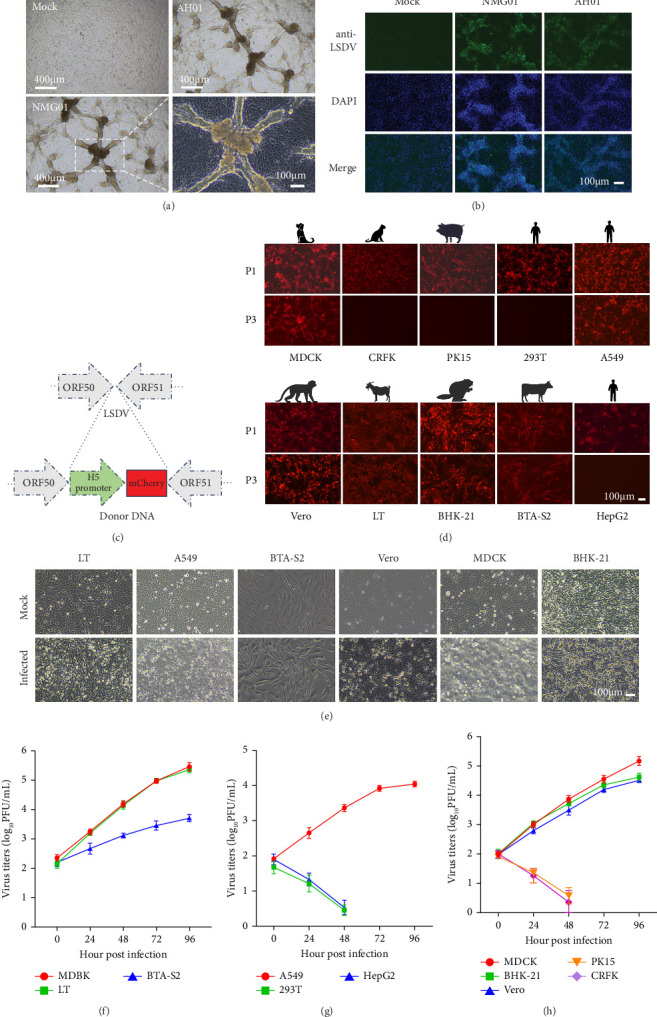
Isolation and identification of LSDV. (a) Characteristic CPE induced by NMG01 strain and AH01 strain in MDBK cell line. (b) IFA for the detection of NMG01 strain and AH01 strain. (c) Construction strategy of the NMG01-mCherry reporter strain. (d) Serial passage characteristics of NMG01-mCherry strain in diverse cell lines. (e) Characteristic CPE induced by LSDV NMG01-mCherry strain in different cell lines. (f) Growth curves of the NMG01 strain in bovine and ovine cell lines. (g) Growth curves of the NMG01-mCherry strain in human cell lines. (h) Growth curves of the NMG01-mCherry strain in canine, feline, hamster, monkey, and porcine cell lines.

After two consecutive passages, samples from two distinct sources (Inner Mongolia and Anhui) caused MDBK cells to exhibit the typical CPE of LSDV, which is mainly typified by lump-like cell aggregation (Figure 1a). IFA using LSDV polyclonal antibodies demonstrated complete overlap between fluorescent signals and CPE lesions (Figure 1b), confirming successful virus isolation. To further analyze the proliferation ability of the isolates in different cell lines, we constructed the NMG01-mCherry strain based on the strategy outlined in Figure 1c. The mCherry gene is driven by the vaccinia virus (VACV) early and late promoter H5 [35]. During the early stages of infection, the expression of fluorescent protein can be used to determine whether LSDV has successfully entered host cells. All tested cell lines infected with NMG01-mCherry strain demonstrated detectable red fluorescence (Figure 1d). However, successful serial passaging of NMG01-mCherry strain was only achieved in MDCK, BHK-21, Vero, LT, A549, and BTA-S2 cell lines, but not in 293T, PK-15, HepG2, and CRFK cell lines (Figure 1d). These results suggest that the isolate entered all 11 mammalian cell lines tested (Figure 1d), although productive replication was only sustained in six (MDCK, BHK-21, Vero, LT, A549, and BTA-S2). Distinct CPE were observed across permissive cell lines. In LT cells, infection induced pronounced cell rounding, whereas in A549, MDCK, BHK-21, and Vero cells, CPE was mainly characterized by cell aggregation and rounding (Figure 1e). CPE was minimal in BTA-S2 cells (Figure 1e). We next examined the replication kinetics of NMG01-mCherry in different cell types. The virus replicated efficiently in bovine MDBK and BTA-S2 cells, ovine LT cells, canine MDCK cells, monkey Vero cells, hamster BHK-21 cells, and human A549 cells, but not in HepG2, PK-15, 293T, or CRFK cells (Figure 1f–h). Among the permissive cell lines, MDBK and LT supported the highest replication levels, indicating that these two cell lines are more susceptible to LSDV than the other tested cell lines (Figure 1f–h).

### 3.2. Complete Morphogenetic Characteristics of LSDV During Replication

**Figure 2:**
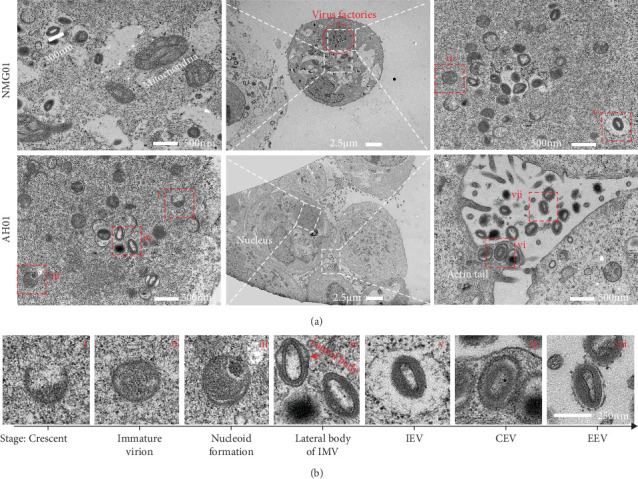
Comprehensive morphogenetic characteristics of LSDV in infected MDBK cells. (a) Transmission electron microscopy (TEM) of NMG01 strain and AH01 strain. (b) Stages of LSDV morphogenesis across its replication cycle.

The morphology of some mature virions of LSDV has been characterized, but the complete morphogenetic characteristics of LSDV remain unclear. To investigate the assembly process of viral particles, we examined viral morphogenesis under electron microscopy. The results revealed that mature viral particles in MDBK cells exhibited an oval shape (about 300 nm in diameter), smaller than mitochondria, and contained an inner core wall (Figure 2a). Additionally, we observed a high-density region in the cytoplasm, comparable in size to the nucleus, which contained numerous viral particles at various stages of assembly (Figure 2a). This region represents the viral factory for LSDV production.

The assembly process of LSDV virions within viral factories and MDBK cells is further summarized as follows (Figure 2b): (i) The virus initially forms crescent-shaped membrane structures. (ii) The membrane progressively extends until closure, forming a spherical immature virion. (iii) Before complete membrane enclosure, the nucleoid containing viral genetic material is incorporated. (iv) Subsequently, symmetrical lateral bodies assemble on both sides of the virion, ultimately forming the intracellular mature virion (IMV). (v) The IMV may acquire an additional membrane from the Golgi apparatus or endoplasmic reticulum, transforming into the intracellular enveloped virion (IEV). (vi) The IEV is then transported to the plasma membrane, where fusion with the cell membrane generates the cell-associated enveloped virion (CEV). (vii) CEVs that detach from the cell surface are released as extracellular enveloped virion (EEV). These images demonstrate relatively complete morphological characteristics of LSDV during its life cycle.

### 3.3. Phylogenetic Analysis of Global Reference Strains

**Figure 3:**
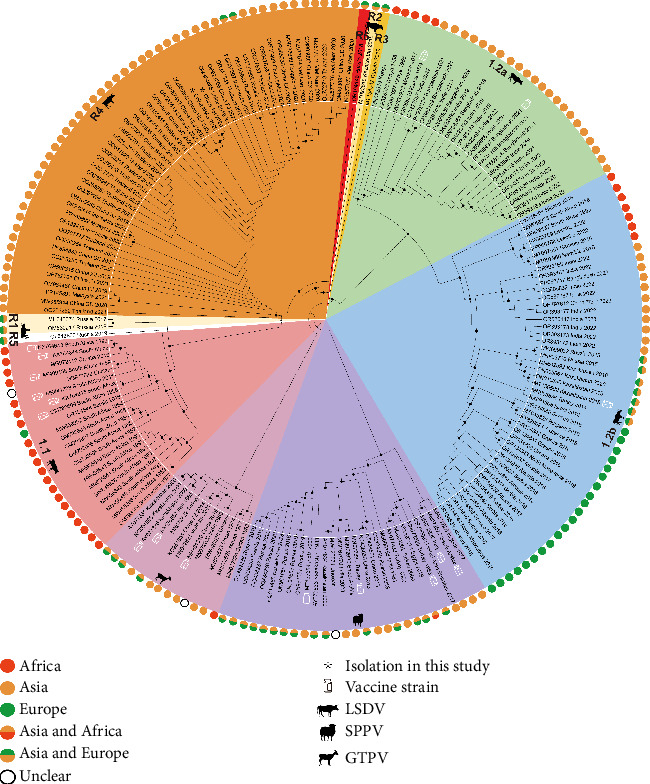
Phylogenetic tree based on whole genome sequences of LSDV. This phylogenetic tree was constructed based on 192 reference sequences of CaPVs. The branch lengths of the tree were adjusted to be ultrametric, ascertaining that the evolutionary time from the root node to all leaf nodes is equal. The nodes marked with black dots have bootstrap values greater than 70%.

We obtained 150 LSDV reference sequences, 13 GTPV sequences, and 27 SPPV sequences from the NCBI database in order to examine the genetic traits of the isolates and global strains. These strains, which were gathered from various locations and eras across the globe, have comparatively comprehensive whole-genome sequencing data. As shown in Figure 3, LSDV strains were categorized into nine clades: 1.1, 1.2a, 1.2b, and R1–R6 in the ML tree based on earlier reports [21, 36, 37]. Among these, clades 1.1, 1.2a, 1.2b, and R1–R5 followed the terminology proposed in earlier studies [35], whereas R6 was represented by a vaccine-like recombinant strain (OR194148) identified in Kurgan, Russia, in 2018, which constitutes a novel recombinant clade [37].

**Figure 4:**
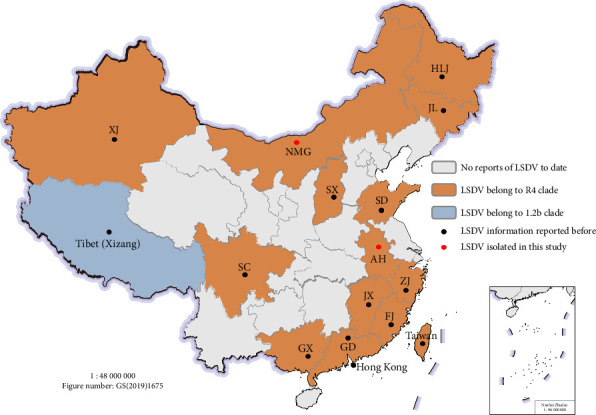
Geographic distribution of Chinese reference strains.

The NMG01 and AH01 strains in this study were found to cluster within clade R4 by phylogenetic analysis, further supporting the dominance of the clade R4 strain in China (Figures 3 and 4). And, all LSDV sequences uploaded from China clustered within clade R4, with the exception of a single sequence from Tibet (Xizang) in 2023 (OR797612), which belonged to Clade 1.2b. The sampling locations of the sequences from China are further indicated in Figure 4. Except for Hong Kong and Taiwan, there have been reports of LSDV outbreaks in 14 provinces on the Chinese Mainland.

### 3.4. Temporal and Geographic Distribution for Global Reference Strains

**Figure 5:**
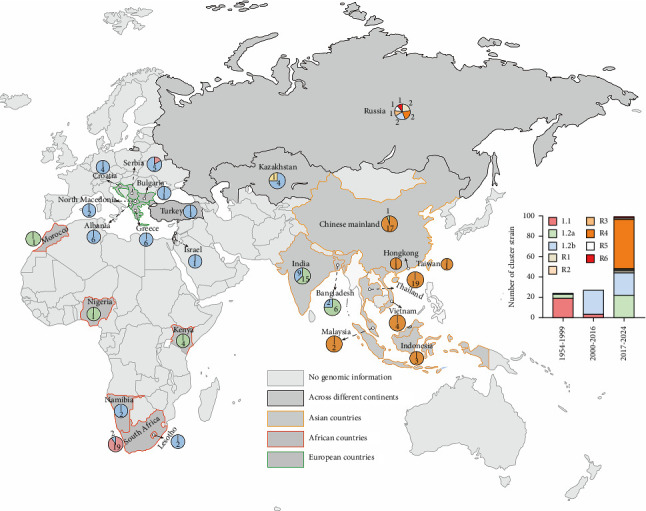
Geographic distribution and temporal statistics of LSDV reference strains. The pie chart illustrates the distribution and proportion of different clade strains across outbreak regions in reference strains. The bar chart statistically shows the proportion of different clade reference strains in three temporal phases.

To better investigate the global epidemiological trends of LSD, we analyzed the time-related information of the reference strains (Figure 5). The Clade 1.1 strain predominated in the database records between 1954 and 1999. The dominant strain from 2000 to 2016 was the Clade 1.2b strain. The Clade R4 strain progressively overtook the Clades 1.2a and 1.2b strains as the most common strain between 2017 and 2024. Furthermore, during this time, there was a notable increase in the number of newly submitted LSDV whole-genome sequences in NCBI database, even surpassing the total cumulatively prior to 2017.

The geographic distribution of the reference strains was also examined (Figure 5). At the continental level, vaccine-like recombinant strains are primarily found in Asia. In addition, the Clade 1.1 strain is primarily distributed in Africa, while the Clade 1.2a strain has been detected throughout Africa, Europe, and Asia. In order to show the continuous evolution of recombination patterns, phylogenetic analysis has further categorized vaccine-like recombinant strains into six different clades (R1–R6) since 2017. The most prevalent strain in Southeast Asia among these is the clade R4 strain.

### 3.5. Detection and Analysis of Recombination Between Different Clade Strains

**Figure 6:**
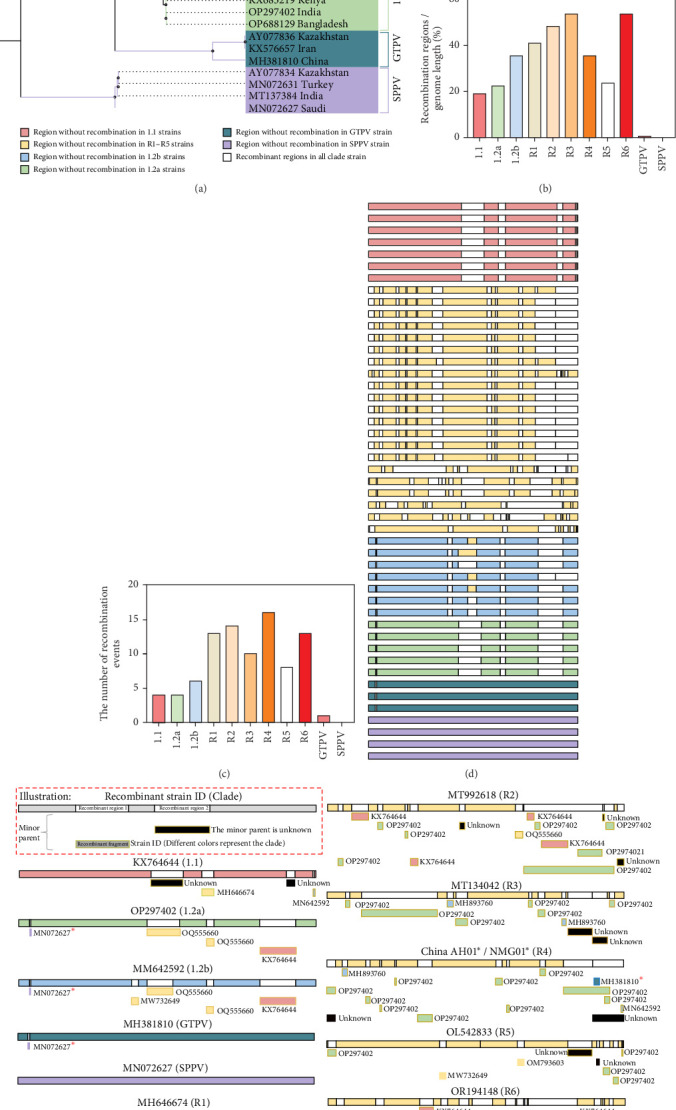
Recombination analysis of reference strains. (a) ML tree reconstructed after removing recombinant regions; nodes marked with black circles have bootstrap values higher than 70%; the tree is drawn to scale, with branch lengths measured in the same units as the evolutionary distances used to infer the phylogenetic relationships. The isolates in this study are marked with a black asterisk. (b) The proportion of recombination regions in the genome calculated based on breakpoint information. (c) The number of recombination events in different clades. (d) Schematic diagram of the reference genome in the reconstructed ML tree: colored bands represent nonrecombinant regions and white bands indicate recombination regions. (e) Recombination events among different clade strains. The red asterisks indicate potential recombination events between LSDV, GTPV, and SPPV. (f) Alignment snapshots around the breakpoint of recombination event 3 (Supporting Information 1: Table S2), showing the recombinant strain (Clade R4, AH01), the minor parents (GTPV vaccine strain AV41 and GTPV field strain MN072621), and the major parent strain (Clade 1.1, MN636841).

Recombination events in sequences can seriously impair the precision of phylogenetic tree construction during phylogenetic analysis [38]. We chose representative strains from various clades, extracted the recombinant nucleotide sequences from these strains, and rebuilt the ML tree in order to remove the impact of recombinant regions (Figure 6a). Among these reference strains, a total of 69 potential recombination events were detected (Supporting Information 1: Table S2). The results showed that OR194148 and MT992618, which had previously clustered in a parallel branch, now formed a monophyletic clade in the reconstructed ML tree (Figure 6a), suggesting that their major parents are similar. Based on the predicted breakpoint information from 69 potential recombination events, we calculated the proportion of recombination regions in the whole genome of each clade strain (Figure 6b). The results showed that recombinant Clades R1–R6 harbored longer recombination regions than Clades 1.1, 1.2a, and 1.2b, with Clade R3 containing the longest recombination regions (Figure 6c).

A schematic representation of the reference genome in the reconstructed ML tree is shown in Figure 6d, which demonstrates that the NMG01 and AH01 strains, as well as other clade R4 strains, share the vast majority of recombination sites. Due to the highly similar recombination sites and patterns observed among strains within the same clade, we describe the specific recombination events of representative strains from each clade (Figure 6e). Vaccine-like recombinant strains showed noticeably more recombination events than classical Clades 1.1 and 1.2 strains (Figure 6c). According to phylogenetic analysis, Clade 1.2 strain was the main recombination partners in recombination events of Clades R1–R6, whereas recombinant Clades R1–R6 strain show a closer genetic affinity to Clade 1.1 strain. These results unequivocally show that Clades 1.1 and 1.2 strains were likely to have undergone genetic recombination to produce the recombinant clade strains.

### 3.6. Vaccine-Recombination in Emerging LSDV Isolates

We further examined recombination risks between heterologous vaccines and LSDV, incorporating three GTPV and four SPPV in the recombination analysis. Statistical results showed that the proportions of recombination regions in the viral genomes of GTPV and SPPV strains were 0.73% and 0.00%, respectively (Figure 6b). In addition, the numbers of potential recombination events identified in GTPV and SPPV strains were 1 and 0, respectively (Figure 6c). Compared with LSDV strains, GTPV and SPPV strains contained the fewest potential recombination events and the shortest recombination regions. However, recombination analysis within the CaPV genus revealed signals of recombination between LSDV and GTPV, LSDV and SPPV as well as GTPV and SPPV. Specifically, in recombination event 3 (Supporting Information 1: Table S2), recombination signals with GTPV as the minor parent were detected in LSDV Clade R4 strains. This event was validated by phylogenetic trees constructed in RDP using the unweighted pair group method with arithmetic mean (UPGMA). In these trees, the recombination regions of Clade R4 strains clustered more closely with the minor parent GTPV vaccine strain, whereas the nonrecombination regions clustered with the representative major parent strain of Clade 1.1 (Supporting Information 2: Figures S1a,b). Similar recombination signals, with SPPV as the minor parent, were also identified in Clades 1.2a and 1.2b (recombination event 11 in Supporting Information 1: Table S2) and in GTPV strains (recombination event 28 in Supporting Information 1: Table S2). These events were also validated by UPGMA phylogenetic trees (Supporting Information 2: Figures S2a,b and S3a,b).

These recombination events among CaPVs were further confirmed using SimPlot software, and the results were consistent with the RDP predictions (Supporting Information 2: Figures S1c–S3c). To determine whether these recombination signals were unique to heterologous vaccine strains, we replaced the vaccine strains with heterologous field strains for validation. The results showed that the same three recombination events were also present in the field strains (Supporting Information 2: Figures S1d–S3d). Representative alignment snapshots around the breakpoint of recombination event 3 (Figure 6f) showed single nucleotide polymorphisms (SNPs) among the recombinant strain (Clade R4, AH01), the minor parents (GTPV vaccine strain AV41 and GTPV field strain MN072621), and the major parent strain (Clade 1.1, MN636841). The results indicated that in the upstream recombination region, AH01 SNPs were highly consistent with those of GTPV (red box in Figure 6f), whereas in the downstream nonrecombination region, AH01 SNPs showed higher similarity to those of Clade 1.1 (yellow box in Figure 6f). These findings further support the occurrence of recombination event 3 (Supporting Information 1: Table S2).

## 4. Discussion

The initial appearance of LSDV recombination strain in Russia in 2017 garnered international attention [10]. This strain then spread throughout Asia and constituted a significant threat to the regional cattle sector. In this study, two LSDV isolates were identified from clinical samples collected during disease outbreaks in China's Inner Mongolia and Anhui Regions. We examined the biological traits of LSDV, such as cellular tropism and morphogenesis, based on the isolates. We conducted thorough analyses of the virus's current epidemic trends, genetic evolution relationship, and recombination patterns by integrating global CaPV reference strains. This study deepens our knowledge of the biological characteristics of LSDV and may contribute to the development of LSD control and prevention strategies.

Only certain cell types can allow for the effective proliferation of LSDV [13], suggesting that LSDV's cellular tropism is rather limited. Nonetheless, the NMG01-mCherry strain demonstrated exceptionally high entry efficiency across all cross-species cell lines that were tested, including nonpermissive cells, indicating that LSDV has a strong capacity to enter mammalian cells. For many viruses, entry typically begins with adhesion to host cell surface proteins, carbohydrates, or lipids, followed by receptor-mediated signaling that triggers internalization into the cytoplasm [39]. To date, no specific host receptors mediating LSDV attachment and internalization have been identified. Previous studies have demonstrated that LSDV predominantly enters MDBK cells via macropinocytosis [40], a nonspecific endocytic pathway that does not rely on receptor-ligand interactions [41]. This indicates that LSDV may not encounter significant cell type restrictions during internalization. Nonetheless, whether receptor-mediated adhesion occurs prior to entry and whether alternative entry pathways exist remain to be further investigated. In addition, our findings showed that both A549 and MDCK cells were permissive for LSDV replication. However, under natural conditions, the likelihood of cross-species infection in humans or dogs appears to be extremely low. First, no natural LSDV infections have been documented in these species to date. Second, members of the Poxviridae family are generally characterized by strict host range restrictions. Moreover, while some studies have confirmed LSDV replication in A549 cells [13], others have reported that replication could not be detected in this cell line [42]. Therefore, as this study was limited to two isolates, the observed cellular tropism may not be fully representative of all LSDV strains.

The life cycle of poxviruses is generally conserved, and IMV and EEV are its two main infectious forms [43]. These two forms of LSDV have been verified by earlier research [44]. In this study, we identified two additional intermediate morphogenetic forms during the life cycle of LSDV: IEV and CEV. Building upon earlier studies on other poxviruses, we logically outlined a reasonably comprehensive morphogenetic process for LSDV. Overall, LSDV shares considerable morphogenetic similarities with VACV [45], which may be attributed to the fact that LSDV encodes at least 30 homologs of poxvirus proteins known to be structural components or involved in virion morphogenesis and assembly. According to the VACV counterpart, different virion types have specific functions during infection: IMV primarily mediate interhost transmission [46], CEV facilitate direct cell-to-cell spread [47], and EEV promote systemic dissemination within the host [48]. Given the striking similarity in virion assembly between LSDV and VACV, the four forms of LSDV may use similar infection strategies.

Since China's first LSDV outbreak in 2019, the epidemic has swiftly spread to multiple provinces [49–51]. This study supplemented near-complete genomic information from Anhui and Inner Mongolia, confirming that recombinant Clade R4 strains are the predominant clade in China. Notably, one Clade 1.2 strain was identified in the first outbreak in the Xizang border region, marking the first report in China of a strain outside the Clade R4 [52]. Border regions are high-risk areas for the cross-border transmission of LSDV, due to the virus's ability to spread via airborne or through arthropod vectors over long distances [49, 53]. Improved epidemiological surveillance in these areas is necessary to lower this risk. From a global standpoint, LSD has evolved from an endemic disease in Africa to a threat on a pandemic scale. As a recently impacted region, Asia shows geographically diverse patterns of transmission: recombinant Clade R4 strains are the main etiological agents in the majority of East Asian and Southeast Asian countries, whereas Clade 1.2 strains are primarily found in South Asian countries, such as Bangladesh [54] and India [14]. The latest surveillance data from the WOAH indicates that LSDV is constantly invading new Asian nations. These recombinant strains are spreading quickly throughout Asia, posing serious biosecurity threats to countries that are not yet impacted. Given that recombinant strains have not spread across Africa and Europe, areas located at continental junctions or intermediate zones may need to increase epidemiological surveillance to prevent the spread of these strains to these continents.

Currently, the main method for preventing and controlling LSD is vaccination with live attenuated vaccines. Nonetheless, genetic recombination between vaccine and field strains is a potential risk factor that warrants further evaluation, and direct causality has not yet been established [55, 56]. In the recombination analysis, several events (e.g., Events 36 and 61) suggested that some strains from clades 1.1, 1.2a, or 1.2b may have originated through recombination with strains from recombinant clades, or that certain recombinant strains arose from recombination among Clades R1–R6. Considering the sampling times of the strains, these recombination events appear to be implausible. This outcome is more likely to reflect representative parental sequences identified by the software rather than the actual historical parents. Since the true ancestral strains may not have been sampled or included in the analysis, and because the RDP method does not incorporate temporal constraints, such situations can arise.

We also detected signals of recombination between LSDV and either SPPV or GTPV. Viral recombination requires the simultaneous infection of the same host cell by two or more viruses. Our cellular tropism experiments demonstrated that LSDV can efficiently invade a wide range of cell types, a property that may also be shared by SPPV and GTPV. Although GTPV and SPPV cannot infect cattle under natural conditions, they are capable of replicating in certain bovine-derived cell lines [57]. Therefore, it is theoretically possible that recombination between LSDV and heterologous vaccine strains could occur when mosquitoes carrying LSDV bite cattle at or near the site of heterologous vaccination. Furthermore, it has been reported that the Lumpivax vaccine used during mass vaccination campaigns in Kazakhstan may have been a mixture of multiple CaPV strains, including GTPV [21], which could further increase the likelihood of interspecies recombination among CaPVs. In this study, we identified three recombination events involving heterologous strains. However, the recombination features observed at these sites were also present in GTPV or SPPV field strains, making it difficult to determine whether these events originated from heterologous vaccine strains or from field strains. In recombination event 3, all progeny viruses clustered within Clade R4, a clade that has only emerged in recent years, suggesting that this event may have occurred relatively recently. Notably, the GTPV vaccine strain has been adopted by many countries, including China, for the prevention and control of LSDV outbreaks, theoretically providing more opportunities for recombination between GTPV vaccine strains and LSDV. Taken together, the available evidence indicates that recombination between LSDV and SPPV or GTPV vaccine strains is possible. While heterologous vaccines have not been associated with field outbreaks, we recommend continued monitoring to ensure that any recombination events do not compromise protective efficacy.

In conclusion, two vaccine-like recombinant strains of LSDV were successfully isolated from outbreaks in Anhui and Inner Mongolia, China. Analysis of their cellular tropism and morphological characteristics revealed that LSDV possesses the ability to enter a wide range of mammalian cell types, and, the complete morphogenetic process of LSDV virions was characterized for the first time. Phylogenetic analysis incorporating global reference strains indicated an accelerated expansion of LSD outbreaks in recent years, with recombinant strains of Clade R4 currently dominating in Asia. Recombination analysis further suggested that heterologous vaccines may contribute only marginally to LSDV recombination. Collectively, these findings expand our understanding of the cellular tropism and morphogenesis of LSDV and provide new insights into its global molecular epidemiology.

## Data Availability

The data that support the findings of this study are available from the corresponding author upon reasonable request.

## Supporting Information

Supporting Information 1 Table S1: Reference sequence information in this study. Table S2: Recombination detection results. Table S3: Primers used in this study.

Supporting Information 2 Figure S1: Phylogenetic tree analysis indicates that GTPV may act as a minor parent for LSDV Clade R4 strains. Figure S2: Phylogenetic tree analysis indicates that SPPV may act as a minor parent for LSDV Clade 1.2 strains. Figure S3: Phylogenetic tree analysis indicates that SPPV may act as a minor parent for GTPV.
