# Supplementary material for: Phylogenetic Analysis, Morphological Characteristics, and Cellular Tropism of Vaccine-Like Recombinant Strains of Lumpy Skin Disease Virus in China
Source: Transbound Emerg Dis. 2025 Dec 2;2025:2900359. doi: 10.1155/tbed/2900359 (PMC12688635; doi:10.1155/tbed/2900359)
Supplement: Supporting Information 2 — Figure S1: Phylogenetic tree analysis indicates that GTPV may act as a minor parent for LSDV Clade R4 strains. Figure S2: Phylogenetic tree analysis indicates that SPPV may act as a minor parent for LSDV Clade 1.2 strains. Figure S3: Phylogenetic tree analysis indicates that SPPV may act as a minor parent for GTPV. [file 2900359.f2.docx]

# Supplementary Material

**Phylogenetic analysis, morphological characteristics, and cellular tropism of vaccine-like recombinant strains of lumpy skin disease virus in China**

Zuxin Gong^1^, Jinming Li^2^, Yanli Zou^2^, Jiaqi Dai^1^, Shan Liu^2^, Lin Li^2^, Chunyan Feng^3^, Fanqi Sun^1^, Xin Li^1^, Chenchen Liu^1^, Zhiliang Wang^2^ *, Gongguan Liu^1^ * and Zhen Yang^1^ *

# Supplementary Figures


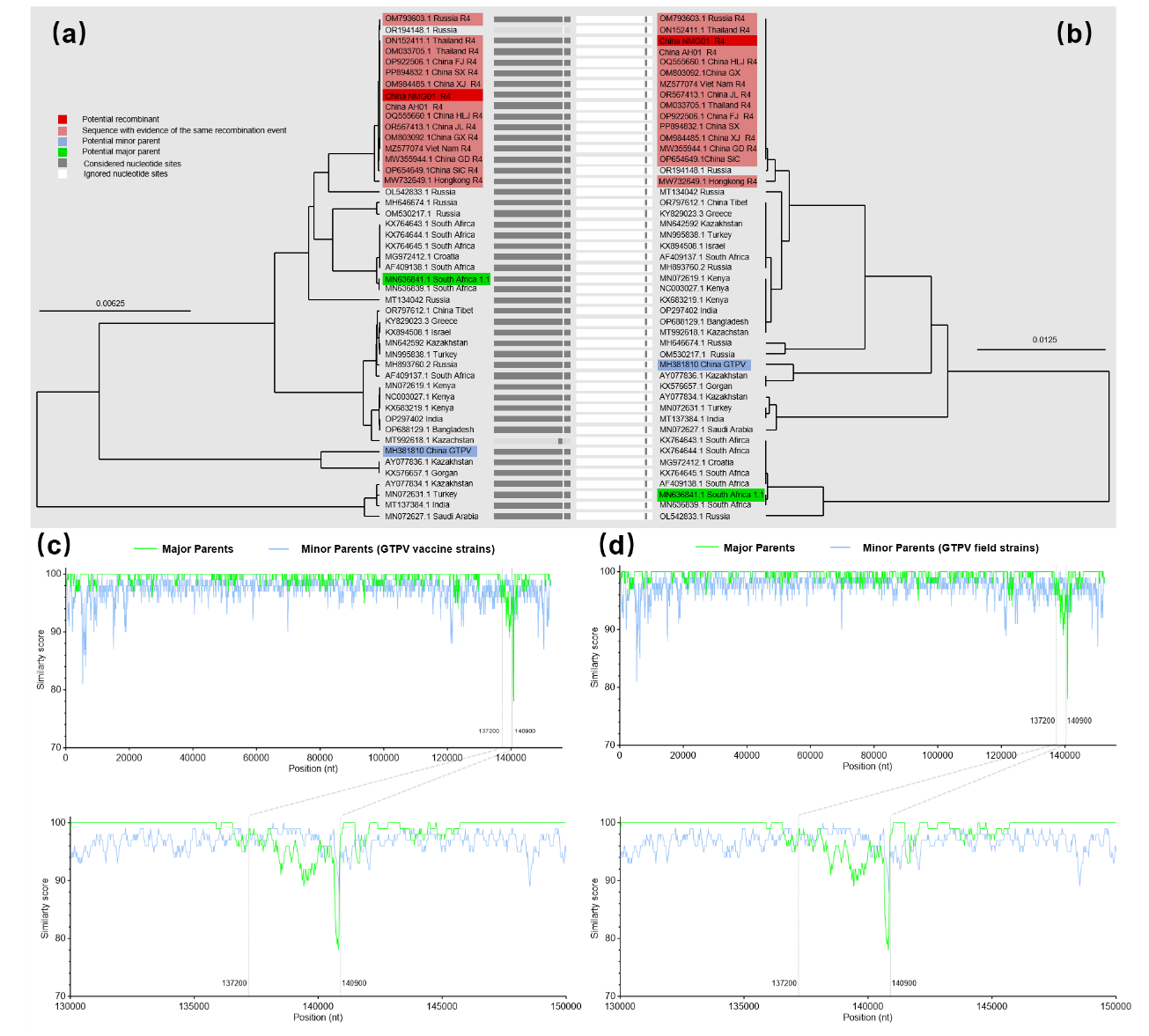


**Figure S1** Phylogenetic tree analysis indicates that GTPV may act as a minor parent for LSDV clade R4 strains. (a) Phylogenetic trees were constructed based on recombination regions (nucleotide sites 1–137238 and 140350–152405) using the Unweighted Pair Group Method with Arithmetic Mean (UPGMA) in RDP4. (b) Phylogenetic trees were constructed based on non-recombination regions (nucleotide sites 137239–140349) using the same method. (c) Similarity plots were generated with SimPlot software between the genomes of the recombinant group strains (clade R4: OP654649, OM793603, ON152411, NMG01, AH01, OQ555660, OM803092, MZ577074, OR567413, OM033705, OP922506, PP894832, OM984485, MW355944, MW732649) and those of the major parent group (clade 1.1: MN636841, KX764643, KX764644, MG972412, KX764645, AF409138, MN636839; clade R1: MH646674, OM530217) or the minor parent group (GTPV vaccine strains: MH381810, AY077836, KX576657). (d) Similarity plots were generated with SimPlot software using GTPV field strains (KC951854, MN072624, NC004003) as the minor parent group in comparison with the recombinant group strains.


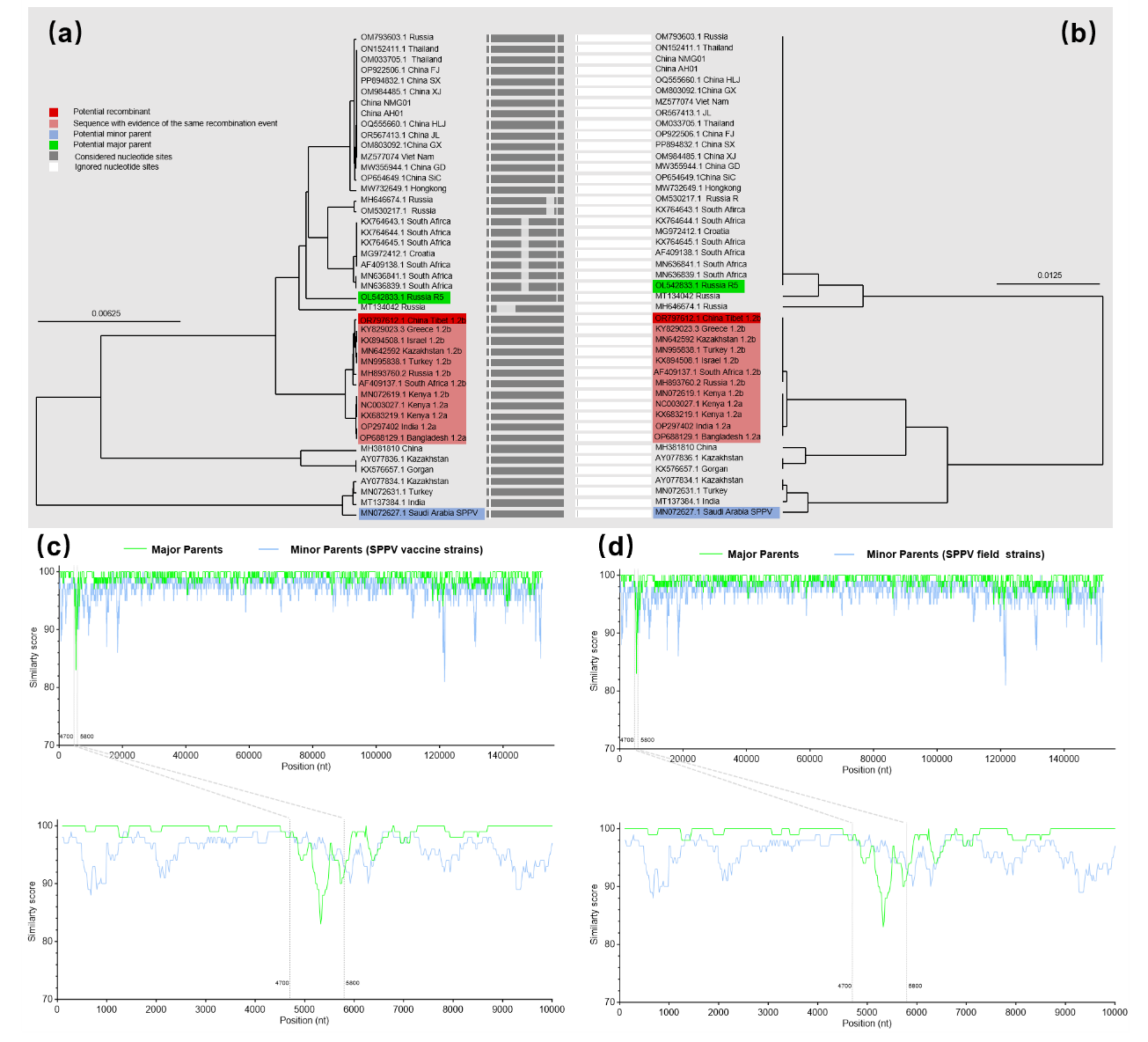


**Figure S2** Phylogenetic tree analysis indicates that SPPV may act as a minor parent for LSDV clade 1.2a or 1.2b strains. (a) Phylogenetic trees were constructed based on recombination regions (nucleotide sites 1–5422 and 6373–152405) using the UPGMA in RDP4. (b) Phylogenetic trees were constructed based on non-recombination regions (nucleotide sites 5423–6372) using the same method. (c) Similarity plots were generated with SimPlot software between the genomes of the recombinant group strains (clade 1.2a: MN072619, NC003027, KX683219, OP297402, OP688129; clade 1.2b: OR797612, KY829023, MN642592, MN995838, KX894508, AF409137, MH893760) and those of the major parent group (clade 1.1: KX764643, KX764644, MG972412, KX764645, AF409138, MN636841, MN636839; clade R3: MT134042; clade R4: OM793603, ON152411, PQ682462, PQ682461, OQ555660, OM803092, MZ577074, OR567413, OM033705, OP922506, PP894832, OM984485, MW355944, OP654649, MW732649; clade R5: OL542833) or the minor parent group (SPPV vaccine strains: MN072627, AY077834, MN072631, MT137384). (d) Similarity plots were generated with SimPlot software between the recombinant group strains and the minor parent group represented by SPPV field strains (OR239060, MW167070, KT438551, KT438550, MN072630, MN072628, MG000156, ON961656, ON961657, OQ434235, OQ434238, OQ434239, OQ434237, OQ434236, AY077834, ON961655, PQ014465, NC004002, AY077832, AY077833, MW167071).


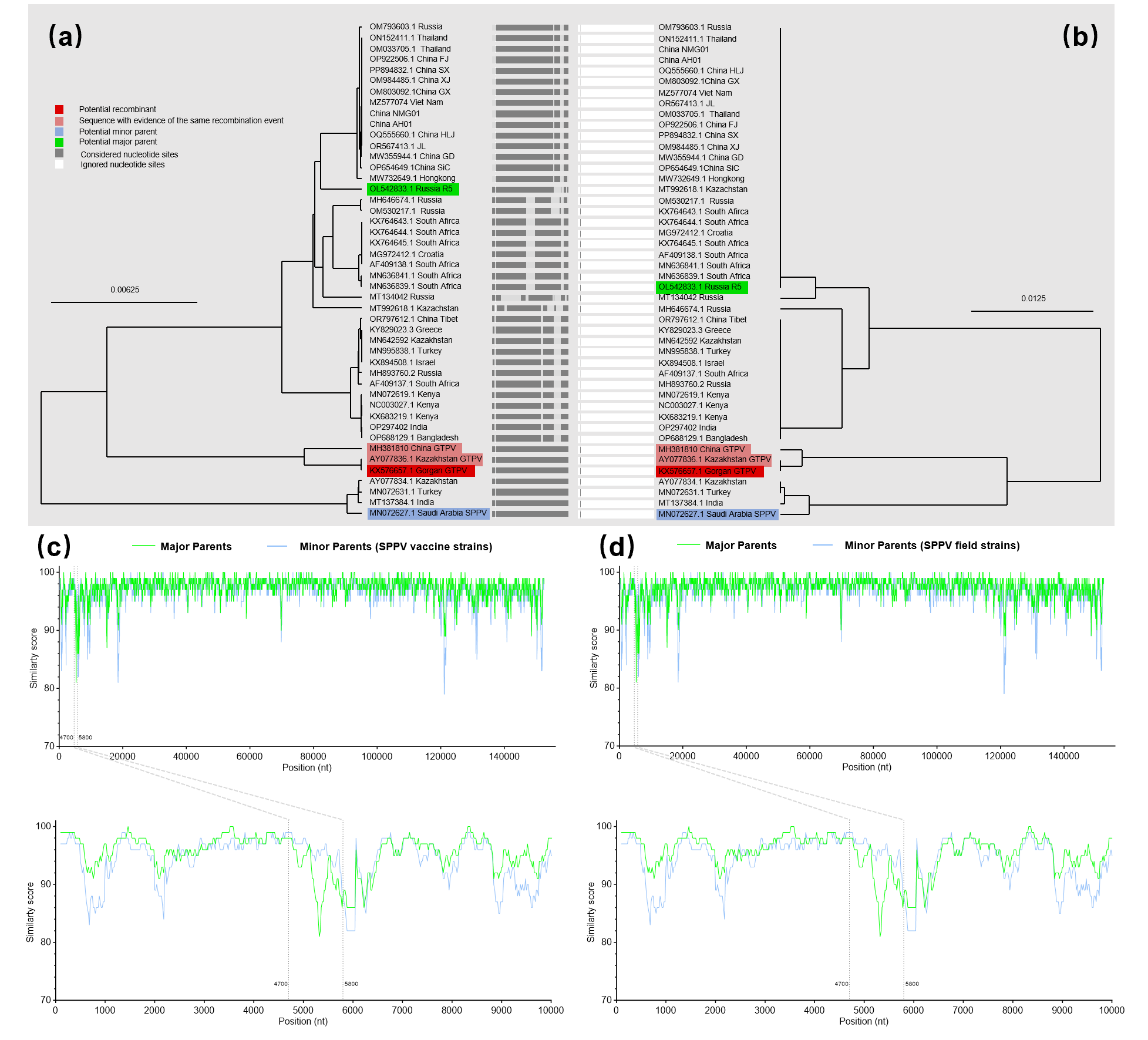


**Figure S3** Phylogenetic tree analysis indicates that SPPV may act as a minor parent for GTPV strains. (a) Phylogenetic trees were constructed based on recombination regions (nucleotide sites 1–5264 and 6406–152405) using the Unweighted Pair Group Method with Arithmetic Mean (UPGMA) in RDP4. (b) Phylogenetic trees were constructed based on non-recombination regions (nucleotide sites 5265–6405) using the same method. (c) Similarity plots were generated with SimPlot software between the genomes of the recombinant group strains (GTPV vaccine strains: MH381810, AY077836, KX576657) and those of the major parent group (clade 1.1: KX764643, KX764644, MG972412, KX764645, AF409138, MN636841, MN636839; clade R3: MT134042; clade R4: OM793603, ON152411, PQ682462, PQ682461, OQ555660, OM803092, MZ577074, OR567413, OM033705, OP922506, PP894832, OM984485, MW355944, OP654649, MW732649; clade R5: OL542833) or the minor parent group (SPPV vaccine strains: MN072627, AY077834, MN072631, MT137384). (d) Similarity plots were generated with SimPlot software between the recombinant group strains and the minor parent group represented by SPPV field strains (OR239060, MW167070, KT438551, KT438550, MN072630, MN072628, MG000156, ON961656, ON961657, OQ434235, OQ434238, OQ434239, OQ434237, OQ434236, AY077834, ON961655, PQ014465, NC004002, AY077832, AY077833, MW167071).
